# MiR-300 Alleviates Cell Proliferation and Migration and Facilitates Cell Apoptosis by Targeting c-Met in Gastric Cancer

**DOI:** 10.1155/2022/6167554

**Published:** 2022-04-04

**Authors:** Xiaoyan Wang, Lian He, Yan Wang, Yunyun Liu, Yuxin Wang, Dandan Chen, Dandan Gong, Yu Fan

**Affiliations:** ^1^Department of Gastroenterology, The Affiliated Suqian First People's Hospital of Nanjing Medical University, Suqian, Jiangsu 223800, China; ^2^Cancer Institute, The Affiliated people's Hospital of Jiangsu University, Zhenjiang, Jiangsu 212002, China

## Abstract

c-Met is a potent oncogene, whose aberrant activation has not been fully clarified. In this study, we discover the biological function of miR-300 in gastric cancer (GC) carcinogenesis and the underlying mechanism. The overexpression, oncogenic functions, and survival analysis of c-Met in GC tissues and cells were firstly determined. miRNAs that potentially targets c-Met were then predicted by bioinformatics. The expression levels of candidate miR-300 in GC tissue pairs were investigated. Pearson analysis revealed a negative relation between miR-300 and c-Met expressions. miR-300 and c-Met expression levels were determined in three GC cell lines (MKN-45, SGC-7901, and AGS) as well. Reduced miR-300 led to increase c-Met levels. Luciferase report assay demonstrated a direct binding site of miR-300 in the 3' untranslated region (3′UTR) of c-Met. Finally, the regulatory role of miR-300 on MKN-45 cells was studied by cell proliferation, migration, and apoptosis assays. Overexpression of miR-300 attenuated viability and migration and accelerated apoptosis in MKN-45. We also induced a rescue experiment with c-Met overexpression plasmid and finally proved that miR-300 exerted a suppressing role on MKN-45 proliferation and migration but promoted MKN-45 apoptosis by directly inhibiting c-Met. This study provides a novel insight into the targeted drug development for GC therapies.

## 1. Introduction

Gastric cancer (GC) is one of the most frequently diagnosed malignancies in the digestive system and the second leading cause of cancer death in the world [1]. Particularly, the morbidity of GC has been increasingly rising for Asian people [2]. Although early GC screening has become more and more widely applied nowadays, current GC therapies are barely satisfactory. The GC mortality still remains high, which causes heavy economic and social burden. Thereby, it is necessary to identify the etiology and pathogenesis of GC and develop novel antitumor therapies.

microRNAs (miRNAs) are highly conserved noncoding RNAs of approximately 22 nucleotides, which can inhibit protein translation or induce messenger RNA (mRNA) degradation by targeting mRNAs in the 3' untranslated region (3′UTR) [3]. As important posttranscriptional regulators, miRNAs regulate multiple cellular activities such as differentiation, proliferation, migration, and apoptosis, so dysregulations of miRNAs contribute to various human diseases including GC [4]. For instance, miR-125b, miR-199a, and miR-100 were the most significant biomarkers for GC progression [5]. miR-140-5p abrogated the growth and invasiveness of GC by targeting YES proto-oncogene 1 (YES1), [6]. miR-34a could be a potent prognostic factor for GC therapy, and in vivo delivery of miR-34a significantly delayed tumor growth in GC mouse models [7]. However, despite many researches, the role of miRNAs in GC carcinogenesis is still not fully understood. This research field deserves further investigations.

C-Met is a receptor-tyrosine protein kinase encoded by the proto-oncogene MET, also known as Met and HGFR (hepatocyte growth factor receptor). HGF is the only high-affinity ligand of c-Met. Under physiological conditions, the HGF/c-Met signaling pathway participates in early embryogenesis development, wound healing, and organ regeneration [8]. In the tumorigenesis, c-Met is aberrantly overexpressed, activated, or mutated. In gastrointestinal tumors, both c-MET and HGF are highly overexpressed, which has been associated with the inferior clinical outcomes of patients with GC. According to previous studies, the MET mRNA amplification and c-MET protein overexpression were discovered in 20–30% and 40–70% of tissue samples from GC patients, respectively [8]. This nonidentity implies that posttranscriptional regulation exists in the c-Met expression during GC development.

In this study, we hypothesized that miRNAs could be the upstream regulators of c-Met and further investigated the role of miRNAs in the GC progression.

## 2. Materials and Methods

### 2.1. Tissues and Cells

Tumor tissues and adjacent tissues were surgically acquired from the First People's Hospital of Suqian and immediately preserved in liquid nitrogen. The patients have been fully informed, and the Ethics Committee of the First People's Hospital of Suqian has approved this research. Immunohistochemistry (IHC) staining was served by the Servicebio Company (Wuhan, China). All the human GC cell lines GFS-1, MKN-7, NC1-N81, AGS, SGC-7901, MKN-45, MGC-803, and HGC-27 were purchased from the Shanghai Institute of Cell Biology, Chinese Academy of Sciences (Shanghai, China). According to the instructions, the cells were cultured in the corresponding culture medium supplemented with 10% fetal bovine serum (FBS, Gibco, USA) in a water-saturated atmosphere of 5% CO2 at 37°C. Lipofectamine 2000 (Invitrogen) was used to conduct cell transfection, followed by detection of transfection efficacy at 24–48 h.

### 2.2. RNA and Protein Extraction

Total RNA and protein were extracted with Trizol reagent (Sigma, USA) or RIPA lysis buffer (Beyotime, China) supplemented with PMSF (Beyotime, China), respectively. NanoDrop (Thermo, USA) and BCA protein assay kit (Thermo Scientific, USA) were used to quantify RNA or protein concentrations.

### 2.3. Quantitative RT-PCR (qRT-PCR)

AMV reverse transcriptase (TaKaRa, China) was used to reverse-transcribe the total RNA. Subsequently, miRNA and mRNA were quantified with TaqMan miRNA probes (Applied Biosystems, USA) or SYBR Green dye (Ambion, USA), respectively. The cycle threshold (CT) was acquired using fixed settings. U6 or GAPDH was used as an internal control. The relative levels of miRNA or mRNA were calculated using the equation 2–△△CT. qPCR primers were as the following: c-Met FP: 5′-CGACAGCTGACTTGCTGAGA-3′, c-Met RP: 5′-AGGTTTATCTTTCGGTGCCCA-3′; GAPDH FP: 5′-GATATTGTTGACATCAATGAC-3′, GAPDH RP: 5′-TTGATTTTGGAGGGATCTCG-3′.

### 2.4. Western Blot (WB)

Proteins were loaded and separated by SDS-PAGE electrophoresis (Bio-Rad, USA) and then transferred onto the polyvinylidene fluoride (PVDF) membranes (Millipore, USA). After being blocked by 5% skim milk for 2 h, the membranes were incubated with primary antibodies at 37°C for 2 h and secondary antibodies for 1 h at room temperature. Bands were exposed and analyzed by ImageJ Software (USA). Involved antibodies were purchased from Cell Signaling Technology (USA).

### 2.5. MiR-300 Overexpression and Knockdown

miR-300 was overexpressed or knocked down by transfecting GC cells with miR-300 mimic or inhibitor (RiboBio, China), respectively. Lipofectamine 2000 (Invitrogen, USA) was used to conduct the transfection following the manufacturer's instructions. Cell culture medium was changed to the medium supplemented with 2% FBS at 4 h after transfection.

### 2.6. C-Met Knockdown and Overexpression

c-Met overexpression plasmid was designed to express the full-length open reading frame (ORF) of the human c-Met gene (Genescript, China). An empty plasmid was used as the negative control (control plasmid, Genescript, China). c-Met siRNA was synthesized to silence c-Met (GenePharma, China). A scrambled siRNA was used as the control (GenePharma, China). c-Met plasmid and c-Met siRNA were transfected into GC cells using Lipofectamine 2000.

### 2.7. Luciferase Reporter Assay

c-Met 3′UTR was inserted into pMIR-report vectors (Ambion, USA) to construct a wild-type luciferase plasmid. The miR-300 binding site was mutated to yield a mutant luciferase plasmid. For luciferase reporter assays, MKN-45, SGC-7901 and AGS cells were co-transfected with a luciferase reporter plasmid, a *β*-galactosidase (*β*-gal) expression plasmid (Ambion, USA), pre-miR-300, and anti-miR-300. Luciferase activity was tested by a luciferase assay kit (Promega Madison, USA).

### 2.8. Cell Proliferation, Migration, and Apoptosis Assays

MKN-45 cells were transfected with RNAs or plasmids. For the proliferation assay, cells were reseeded in 96-well plates after transfection. Cell Counting Kit-8 (Dojindo, Japan) was used to measure the absorbance at a wavelength of 450 nm at 12, 24, 36, 48, 60, and 72 h. Millipore 24-well plates containing an 8-*μ*m pore membrane were used to conduct the migration assay. After transfection, cells were reseeded in the upper compartment filled by FBS-free culture medium, while 10% FBS culture medium was added to the lower compartment. After incubation for 48 h, cells were fixed with 4% paraformaldehyde for 15 min and then stained with 0.1% crystal violet in methanol for 15 min. Cells on the upper surface (nonmigrant) were scraped off using a cotton swab after washing. The lower surfaces with migrant cells were captured and counted. For the apoptosis assay, LPS (Sigma, USA) was added (400 ng/mL) in FBS-depleted medium for 24 h to induce apoptosis after transfection. Cell apoptosis was assayed by flow cytometric analysis with an Annexin V-FITC/PI staining kit (BD Biosciences, USA) according to the instructions.

### 2.9. Bioinformatics Analyses

TargetScan and miRDB were used to predict candidate miRNAs. Survival analysis was conducted using patient information from the cancer genome atlas (TCGA) database.

### 2.10. Statistical Analysis

All experiments were repeated three times, and the data are presented as the mean ± standard deviation using SPSS 18.0 (SPSS, Inc.). One-way ANOVA and post hoc Dunnett's T3 test were performed in order to compare the differences among and between groups, respectively. *P* < 0.05 was considered to indicate a statistically significant result.

## 3. Results

### 3.1. C-Met Is Overexpressed in GC

**Figure 1:**
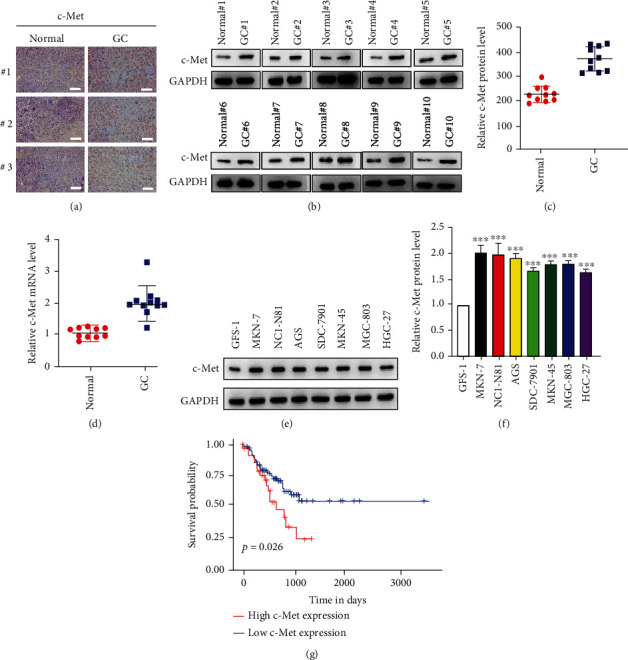
c-Met is upregulated in GC tissues and cell lines. (a) IHC staining of c-Met in GC tissue pairs. Scar bar: 20 *μ*m. (b and c) WB analysis of c-Met protein levels in GC tissue pairs. (d) qRT-PCR analysis of c-Met mRNA levels in GC tissue pairs. (e and f) WB analysis of c-Met levels in GC cell lines. (g) The effect of c-Met expression on overall survival of patients with GC according to the TCGA dataset. *N* = 3, ∗*P* < 0.05; ∗∗*P* < 0.01; ∗∗∗*P* < 0.001.

To detect the c-Met expressions in GC tumor tissues, we performed IHC staining, WB, and qRT-PCR analysis. As shown, both c-Met protein (Figures 1(a)–1(c)) and mRNA (Figure 1(d)) levels were upregulated in GC tumor tissues compared to the adjacent tissues. We also detected c-Met levels in multiple GC cell lines; results show that c-Met is significantly upregulated in GC cell lines compared to the normal gastric epithelial cell GFS-1 (Figures 1(e) and 1(f)). Through Kaplan-Meier analysis, we found that c-Met overexpression leads to worsen the overall survival outcomes in GC patients (Figure 1(g)).

### 3.2. C-Met Promotes the Proliferation and Migration but Reduces the Apoptosis in GC Cells

**Figure 2:**
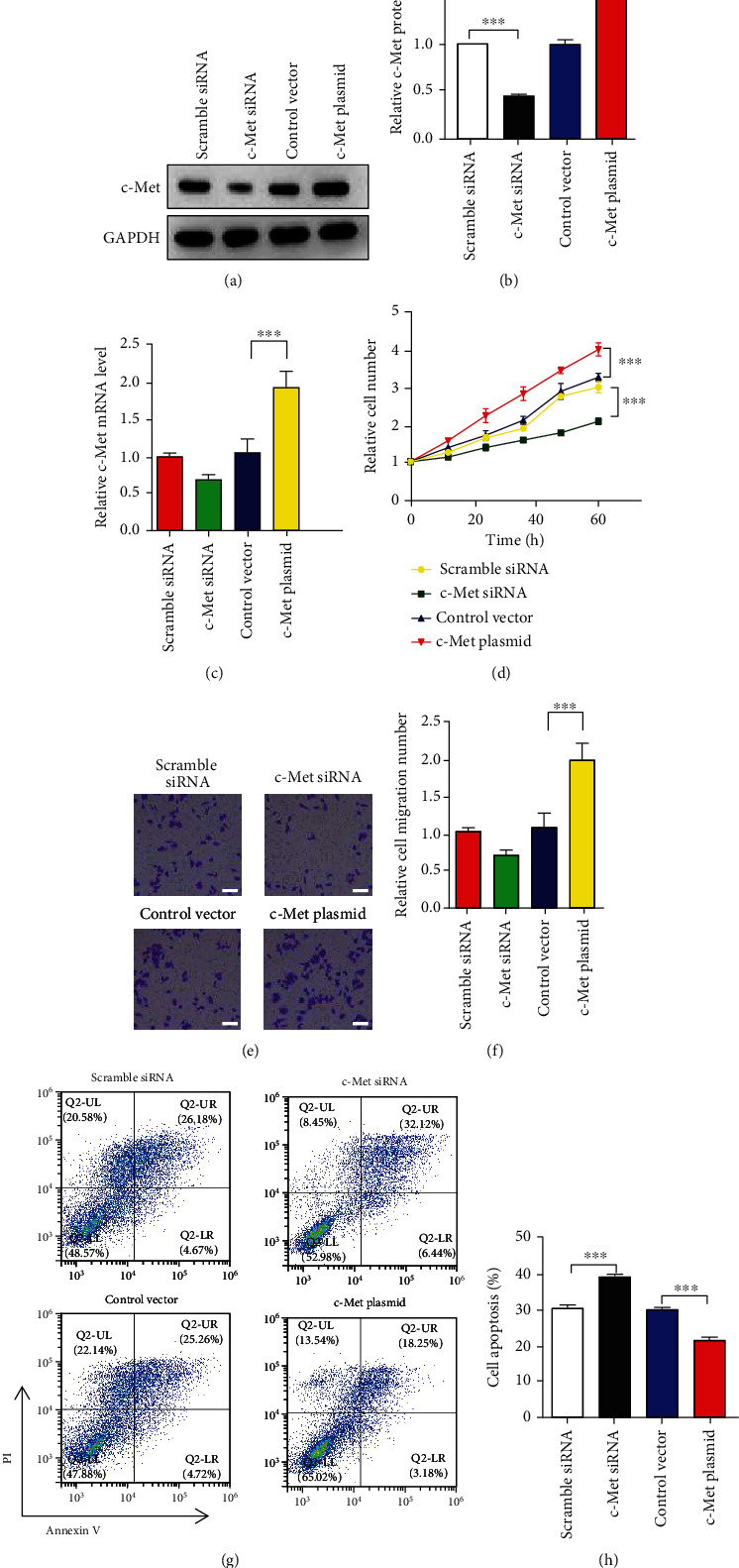
c-Met promotes GC cell proliferation and migration and reduced cell apoptosis. (a) WB analysis of c-Met levels in MKN-45 cell line after transfection with c-Met siRNA or c-Met plasmid. (b–c) qRT-PCR analysis of c-Met levels in MKN-45 cell line after transfection with c-Met siRNA or c-Met plasmid. (d) CCK-8 assay analysis of MKN-45 proliferation after transfection with c-Met siRNA or c-Met plasmid. (e and f) Transwell assay analysis of MKN-45 cell migration after transfection with c-Met siRNA or c-Met plasmid. Scar bar: 20 *μ*m. (g and h) MKN-45 cell apoptosis was assayed by flow cytometric analysis after transfection with c-Met siRNA or c-Met plasmid and treated with LPS. *N* = 3, ∗*P* < 0.05; ∗∗*P* < 0.01; ∗∗∗*P* < 0.001.

Although c-Met is a famous oncogene, we still wonder the specific functions of c-Met in GC cell lines. We next determined the influence of c-Met on GC cell proliferation, migration, and apoptosis in MKN-45 using CCK-8, transwell, and apoptosis assays. As presented, overexpression of c-Met (Figures 2(a)–2(c)) remarkably promoted MKN-45 proliferation (Figure 2(d)) and migration (Figures 2(e) and 2(f)) but reduced MKN-45 apoptosis induced by LPS (400 ng/mL) (Figures 2(g) and 2(h)), and vice versa.

### 3.3. C-Met Is Directly Targeted by miR-300

**Figure 3:**
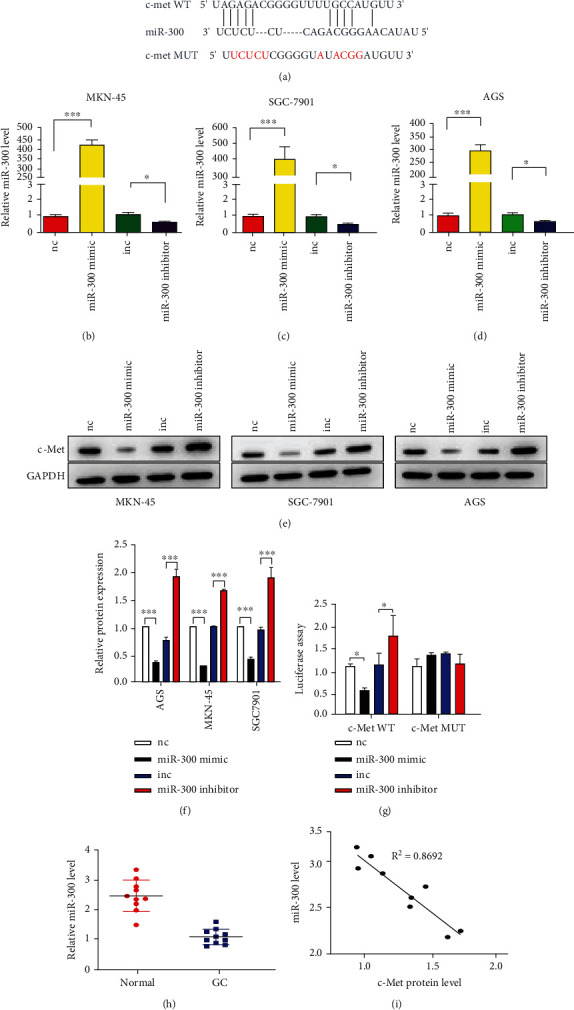
c-Met is a direct target gene of miR-300 in GC. (a) Schematic description of the predicted binding site of miR-300 in the 3′UTR of c-Met gene. (b–d) qRT-PCR analysis of miR-300 levels in MKN-45, SGC-7901, and AGS cells after transfection with miR-300 mimic or inhibitor. (e and f) WB analysis of c-Met protein levels in 3 GC cell lines after transfection with miR-300 mimic or inhibitor. (g) Relative luciferase activities in MKN-45 after transfection with miR-300 mimic or inhibitor. (h) qRT-PCR analysis of miR-300 levels in GC tissue pairs. (i) Pearson analysis of the relationship between miR-300 level and c-Met protein level in GC tissue pairs. *N* = 3, ∗*P* < 0.05; ∗∗*P* < 0.01; ∗∗∗*P* < 0.001.

Because miRNAs are important posttranscriptional regulators of protein-coding genes, we next figured out miRNAs that potentially target c-Met. Through bioinformatic analysis using TargetScan [9] and miRDB [10] databases, we finally focused on miR-300 which has a most probable binding site in the 3′UTR of c-Met (Figure 3(a)). To investigate the potential relation between miR-300 and c-Met expression levels, we downregulated or overexpressed miR-300 in MKN-45, SGC-7901, and AGS cells by transfecting miR-300 mimic and inhibitor. Successful transfections are shown in Figures 3(b)–3(d). Subsequently, c-Met protein levels were significantly suppressed by miR-300 (Figures 3(e) and 3(f)). Besides, the high expression of miR-300 dramatically decreased the luciferase activity, while inhibition of miR-300 increased the fluorescence intensity (Figure 3(g)). Moreover, we tested that miR-300 levels were reduced in GC tumor tissues compared to the adjacent tissues (Figure 3(h)). Pearson analysis revealed an inverse correlation between miR-300 and c-Met protein levels (Figure 3(i)). These results suggested that miR-300 directly targeted c-Met in the 3′UTR region.

### 3.4. MiR-300 Prevents GC Cells Functions by Inhibiting c-Met

**Figure 4:**
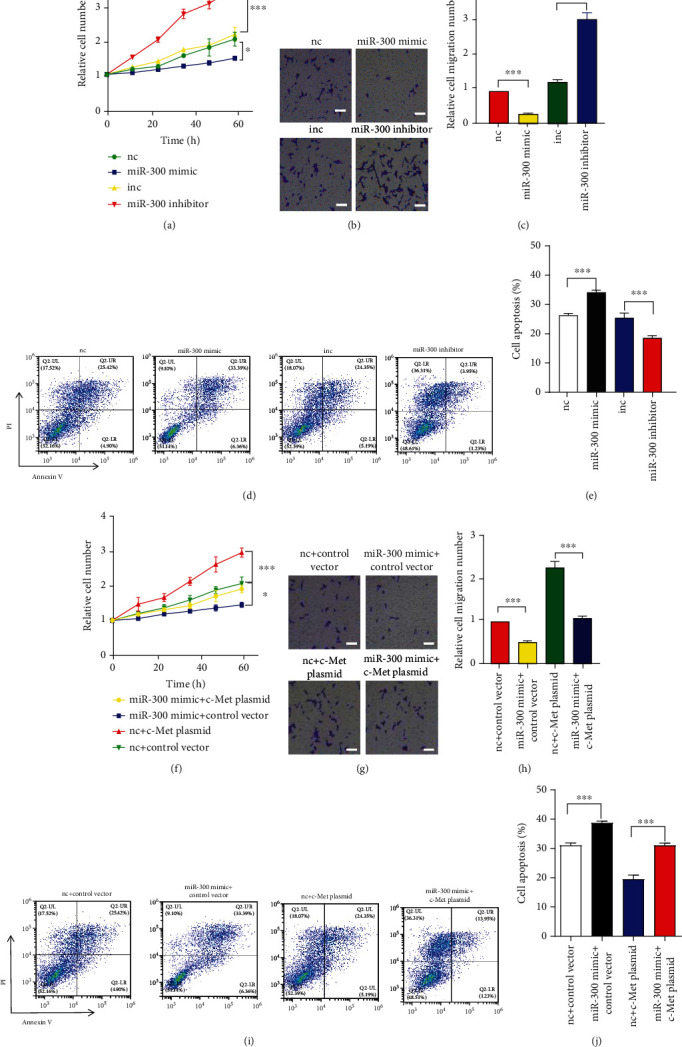
miR-300 suppresses GC cell proliferation and migration and increased cell apoptosis. (a) CCK-8 assay analysis of MKN-45 proliferation after transfection with miR-300 mimic or inhibitor. (b and c) Transwell assay analysis of MKN-45 cell migration after transfection with miR-300 mimic or inhibitor. Scar bar: 20 *μ*m. (d and e) MKN-45 cell apoptosis was assayed by flow cytometric analysis after transfection with miR-300 mimic or inhibitor and treated with LPS. (f) CCK-8 assay analysis of MKN-45 proliferation after co-transfection with miR-300 mimic and c-Met plasmid. (g and h) Transwell assay analysis of MKN-45 cell migration after co-transfection with miR-300 mimic and c-Met plasmid. Scar bar: 20 *μ*m. (i and j) MKN-45 cell apoptosis was assayed by flow cytometric analysis after co-transfection with miR-300 mimic and c-Met plasmid and treated with LPS. *N* = 3, ∗*P* < 0.05; ∗∗*P* < 0.01; ∗∗∗*P* < 0.001.

We further studied the role of miR-300 in MKN-45 by CCK-8, transwell, and apoptosis assays. As shown, miR-300 distinctly inhibited MKN-45 proliferation (Figure 4(a)) and migration (Figures 4(b) and 4(c)), whereas miR-300 could induce MKN-45 apoptosis induced by LPS (400 ng/mL) (Figures 4(d) and 4(e)). These data indicated that miR-300 exerts as a tumor suppressor in GC cells. To study whether miR-300 work through suppressing c-Met, we co-transfected miR-300 and c-Met plasmid into the MKN-45 cell. As a result, c-Met plasmid could reverse the influences of miR-300 on MKN-45 proliferation (Figure 4(f)), migration (Figures 4(g) and 4(h)) and apoptosis induced by LPS (400 ng/mL) (Figures 4(i) and 4(j)).

## 4. Discussion

As an important oncogene, c-Met is overexpressed in various cancers, such as kidney cancer, lung cancer, glioblastoma, colon cancer, esophageal cancer, and gastric cancer [11–14]. Consistent with previous studies, our study demonstrated overexpression of c-Met in GC. And by Kaplan-Meier analysis, it was found that c-Met overexpression leads to poor overall survival outcomes in GC patients. However, the mechanism by which c-Met is abnormally activated in malignant tumors is unclear. Previous studies have focused on the downstream pathways of c-Met, such as phosphatidylinositol-3-kinase/protein-serine-threonine kinase (PI3K/Akt), Jun amino-terminal kinase (JNK)/p38 MAPK cascade, nuclear factor-*κ*B (NF-*κ*B), and sarcoma gene/focal adhesion kinase (SRC/FAK) [15]. Few studies have focused on why c-Met is overexpressed in tumorigenesis. Notably, in this study, we innovatively explored the efficient upstream regulation of c-Met.

As posttranscriptional regulators, miRNAs are widely involved in various pathological and physiological processes, and the abnormal expression of miRNAs is closely related to the occurrence and development of cancer [16]. Previous studies have made great progress in discovering tumor-associated miRNAs, which have greatly advanced our understanding of tumor pathogenesis. For example, in GC development, miR-140-5p [17], miR-29c [18], and miR-139 [19] act as tumor suppressors, while miR-224 [20] promotes GC progression. These studies suggest that miRNAs may be strong regulators involved in GC tumorigenesis. However, this study focused on miR-300. miR-300 expression is reduced in a variety of malignancies, including oral cancer [21], bladder cancer [22], pancreatic cancer [23], and hepatocellular carcinoma (HCC) [24]. However, miR-300 has been little studied in gastric cancer. Here, we found that miR-300 expression levels were also reduced in gastric cancer tissues and that miR-300 inhibited GC cell proliferation and migration and increased apoptosis.

Mechanistically, miR-300 regulates cancer cell behavior by targeting downstream genes. To date, several target genes of miR-300 have been identified. For example, miR-300 targets LEF-1 to inhibit HCC cell proliferation and metastasis [25]; miR-300 targets CRL4B to inhibit the development of pancreatic cancer [23]; miR-300 targets twist to inhibit the transformation of epithelial cells to mesenchymal cells [26]; and miR-300 inhibits the metastasis of osteosarcoma cells by targeting PTTG1 [27]. Our study showed that miR-300 directly targets the 3′UTR region of c-Met, thereby inhibiting the overexpression of c-Met in GC and hindering the function of gastric cancer cells. These findings suggest that miR-300 may be a potential therapeutic target and prognostic factor for GC.

Despite recent advances in therapeutic strategies, GC-related mortality remains high [28]. This is mainly because most patients are diagnosed at an advanced stage or have limited treatment options [29] Therefore, further efforts should be made to find new biomarkers or targeted therapy. At present, although the targeted drugs of c-Met are booming, there are still few effective GC-targeted therapies [30]. miRNA mimics and miRNA inhibitors in preclinical development have shown promise as novel therapeutics [31]. In this case, miR-300 may bring a breakthrough and may be more worthy of attention in the field of drug development.

## 5. Conclusions

In this study, we found that c-Met was up-regulated in GC, and c-Met high expression was associated with poor prognosis in GC patients. The expression of miR-300 in GC was negatively correlated with c-Met protein level. We innovatively demonstrated that miR-300 directly targets the 3′UTR region of c-Met to inhibit the proliferation and migration of gastric cancer cells and promote apoptosis. Our study provides complementary evidence for further elucidation of the molecular mechanisms of GC proliferation and metastasis. Overall, miR-300 may be a promising therapeutic target for gastric cancer. It is necessary to further study the clinical significance and regulatory mechanism of miR-300 in the future to determine its role in the treatment of GC.

## Data Availability

The raw data supporting the conclusions of this article will be made available by the authors, without undue reservation.
